# Valosin-containing protein is a key mediator between autophagic cell death and apoptosis in adult hippocampal neural stem cells following insulin withdrawal

**DOI:** 10.1186/s13041-016-0212-8

**Published:** 2016-03-22

**Authors:** Bo Kyoung Yeo, Caroline Jeeyeon Hong, Kyung Min Chung, Hanwoong Woo, Kyungchan Kim, Seonghee Jung, Eun-Kyoung Kim, Seong-Woon Yu

**Affiliations:** Department of Brain and Cognitive Sciences, Daegu Gyeongbuk Institute of Science and Technology (DGIST), Daegu, 711-873 Republic of Korea; Neurometabolomics Research Center, Daegu Gyeongbuk Institute of Science and Technology (DGIST), Daegu, 711-873 Republic of Korea

**Keywords:** Autophagic cell death, Apoptosis, Adult neural stem cells, Insulin withdrawal, Valosin-containing protein

## Abstract

**Background:**

Programmed cell death (PCD) plays essential roles in the regulation of survival and function of neural stem cells (NSCs). Abnormal regulation of this process is associated with developmental and degenerative neuronal disorders. However, the mechanisms underlying the PCD of NSCs remain largely unknown. Understanding the mechanisms of PCD in NSCs is crucial for exploring therapeutic strategies for the treatment of neurodegenerative diseases.

**Result:**

We have previously reported that adult rat hippocampal neural stem (HCN) cells undergo autophagic cell death (ACD) following insulin withdrawal without apoptotic signs despite their normal apoptotic capabilities. It is unknown how interconnection between ACD and apoptosis is mediated in HCN cells. Valosin-containing protein (VCP) is known to be essential for autophagosome maturation in mammalian cells. VCP is abundantly expressed in HCN cells compared to hippocampal tissue and neurons. Pharmacological and genetic inhibition of VCP at basal state in the presence of insulin modestly impaired autophagic flux, consistent with its known role in autophagosome maturation. Of note, VCP inaction in insulin-deprived HCN cells significantly decreased ACD and down-regulated autophagy initiation signals with robust induction of apoptosis. Overall autophagy level was also substantially reduced, suggesting the novel roles of VCP at initial step of autophagy.

**Conclusion:**

Taken together, these data demonstrate that VCP may play an essential role in the initiation of autophagy and mediation of crosstalk between ACD and apoptosis in HCN cells when autophagy level is high upon insulin withdrawal. This is the first report on the role of VCP in regulation of NSC cell death. Elucidating the mechanism by which VCP regulates the crosstalk of ACD and apoptosis will contribute to understanding the molecular mechanism of PCD in NSCs.

## Background

Programmed cell death (PCD) is a deliberate cellular suicide process. Dysfunction of PCD is implicated in various human diseases, including developmental and neurodegenerative disorders, cancer and autoimmune diseases [1]. PCD can be categorized by morphological criteria [2]. Type I cell death, known as apoptosis, is characterized by cell shrinkage, nuclear condensation, membrane blebbing, mitochondria dysfunction, loss of selectivity in membrane permeabilization, and nuclear DNA fragmentation. Lastly, cells are rapidly eliminated by phagocytosis [3]. Type II PCD refers to autophagic cell death (ACD). Autophagy is a catabolic process that disposes of various cytoplasmic components, including protein aggregates and organelles [4]. The components are sequestered by autophagosomes, which fuse with lysosomes for degradation. This process usually occurs in response to cellular stress to protect the cells. However, prolonged autophagy can cause ACD [5]. Type III cell death, called necrosis, is best defined by abnormal mitochondria morphology, swollen and ruptured cellular membrane, cell lysis and exposure of intracellular content to the extracellular space. Therefore, inflammatory reactions are frequently related with necrosis [6].

Autophagy is the major degradation pathway for long-lived proteins and damaged organelles for clearance or recycling. Autophagosomes deliver cytoplasmic contents to lysosome where autophagosomes become autolysosomes for degradation. Autophagy process takes place in eukaryotic cells from yeast to mammals for cellular remodeling during development as well as starvation, inflammatory reaction and cell death [7, 8]. Majority of the studies on the physiological roles of autophagy suggest that autophagy is a protective process by helping to cope with cellular stress and maintain cellular homeostasis. However, there are reports on the causative role of autophagy in developmental cell death. Cell death of the salivary glands during *Drosophila* development is mediated by autophagy genes (Atg) in a caspase-dependent or independent manner [9, 10]. Also, excessive autophagy in response to stress or injury can cause ACD [11, 12]. However, despite the emerging role of autophagy in regulation of PCD, the underlying mechanisms are poorly understood.

Previously we reported hippocampal neural stem (HCN) cells undergo ACD upon insulin withdrawal [13]. Cell death induced by insulin depletion did not show apoptotic signs. Instead autophagic markers were significantly increased, whereas anti-apoptotic/anti-autophagic proteins, Bcl-2 and Bcl-X_L_, were decreased. Importantly, cell death rate was significantly decreased with knockdown of Atg7 in insulin-deprived HCN cells. Of note, high calpain activity switched the cell death mode from ACD to apoptosis [14]. Interestingly, activation of glycogen synthase kinase-3β (GSK-3β), one of the key signaling molecules in regulation of neuronal apoptosis, also promoted ACD, not apoptosis, in insulin-deprived HCN cells [15]. These data suggest that there is the unique intrinsic cell death program that drives the cell death mode towards ACD rather than apoptosis in HCN cells following insulin withdrawal. Currently, HCN cell death induced by insulin withdrawal is regarded as the most genuine model of ACD in mammals [16].

Valosin-containing protein (VCP)/p97 is a ubiquitously expressed protein belonging to the AAA+ (**A**TPases **A**ssociated with diverse cellular **A**ctivities) protein family with two ATPase domains, D1 and D2 [17]. Following binding of the substrates to the N and C terminal domains, VCP hydrolyses ATP on its ATPase domains. Subsequently, VCP changes its complex formation with distinct interacting proteins or cofactors to exert its multicellular functions [18–25]. Previous studies have reported that VCP is involved in multiple cellular processes, including cell cycle regulation, Golgi biogenesis, nuclear membrane formation, ubiquitin proteasome system (UPS), apoptosis and the autophagosome maturation [17, 26]. Cells with loss of VCP activity failed to undergo autophagosome and lysosome fusion, thereby prevented autophagosome maturation, suggesting the positive regulation of autophagosome maturation by VCP in mammalian cells [27–29]. Mutations in human VCP is associated with the multisystem disease called “inclusion body myopathy associated with Paget’s disease of bone and frontotemporal dementia (IBMPFD)”, which is featured with inclusion bodies in the brain or muscle [28, 30]. Furthermore, depletion or ATPase-inactive mutants of VCP induced apoptosis in several different types of cells [31]. These previous studies prompted us to examine the involvement of VCP in regulation of ACD in HCN cells following insulin withdrawal.

In this study, we report the different actions of VCP depending on the autophagy level. Inactivation of VCP at basal state in the presence of insulin led to mild impairment of autophagy, indicating involvement in autophagosome maturation, as previous reported by others. On the other hand, pharmacological and genetic inhibition of VCP in insulin-deprived HCN cells undergoing high level of autophagy decreased autophagy initiation signaling and reduced ACD, concomitant with robust induction of apoptosis. These results suggest a novel role of VCP in mediation of autophagy, implicating VCP at the early stage of autophagy as well as the late maturation step depending on the autophagy level. Our study, for the first time, reveals the critical role of VCP in crosstalk between ACD and apoptosis, indicating VCP as a distinct regulator in survival and death of neural stem cells (NSCs).

## Results

### VCP is highly expressed in HCN cells and degraded by autophagy following insulin withdrawal

**Fig. 1:**
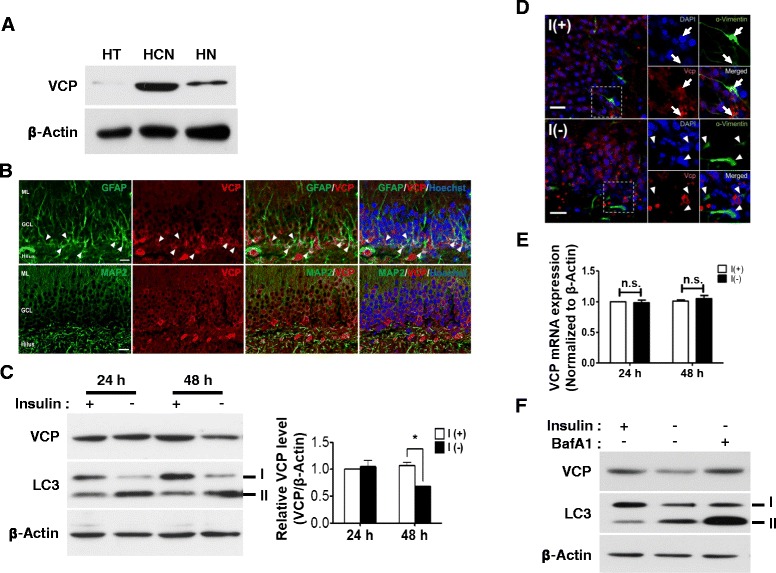
VCP is highly expressed in HCN cells and degraded through autophagy following insulin withdrawal. **a** VCP was abundantly expressed in HCN cells compared with hippocampal tissue prepared from 8-week-old rat and embryonic primary hippocampal neurons (9 days in vitro). **b** VCP was particularly localized in hippocampal neural stem cells in vivo, as indicated by *arrowheads*. GFAP and MAP2 were used as NSC and neuronal markers, respectively. Scale bar, 20 μm. **c** VCP protein level was significantly decreased at 48 h following insulin withdrawal. Quantitative analysis of VCP levels was normalized to β-actin. Quantitative data are represented at the mean ± SD (*n* = 3). Statistical significance was determined with an One-way ANOVA test by Tukey’s multiple comparison test. *p < 0.05. **d** VCP expression levels were decreased in *ex vivo* organotypic hippocampal slice culture following insulin withdrawal for 24 h. *Arrow* indicates the VCP-expressing HCN cells in I(+) condition, while *arrowheads* show the loss of VCP expression in I(-) condition. Scale bar, 20 μm. **e** Expression levels of VCP mRNA were not changed by insulin withdrawal. Quantitative data are represented at the mean ± SD (*n* = 3). n.s. not significant. **f** Degradation of VCP was prevented by Bafilomycin A1 treatment (BafA1, 30 nM for 3 h before harvest) following 48 h insulin withdrawal

Since VCP has not been studied in NSCs before, first, we examined the expression level of VCP in HCN cells. VCP was highly expressed in HCN stem cells in vitro, compared to the total hippocampal tissue derived from 8-week-old rat and embryonic hippocampal primary neurons after 9 days in vitro (Fig. 1a). To further examine whether VCP is abundantly expressed in HCN cells than hippocampal neurons in vivo, we performed immunohistochemical analysis in 8-week-old rat brain tissue, since HCN cells were derived from the adult rat hippocampus of the same age [13]. VCP was highly expressed in the NSCs in the subgranular zone (SGZ) of the dentate gyrus, as revealed by co-localization with glia fibrillary acidic protein (GFAP), a NSC maker in SGZ [32]. VCP was also expressed in the granule neurons of the DG in adult hippocampus, when microtubule-associated protein 2 (MAP2) was used as a neuronal marker [33] (Fig. 1b). The time course analysis of VCP protein and mRNA expression levels was performed following insulin withdrawal. From here, we denote the insulin-containing and insulin-depleted culture conditions as I(+) and I(-), respectively. Consistent with our prior studies [13–15], high level of autophagy was induced in I(-) (Fig. 1c). Autophagy induction was verified by an increase in the level of type II of microtubule-associated protein 1 light chain 3 (LC3-II), since this is the most reliable biochemical marker of autophagy [34–36]. VCP protein level was decreased 48 h following insulin withdrawal (Fig. 1c). To further confirm the decrease of VCP protein level following insulin withdrawal in ex vivo HCN culture, we measured VCP levels in organotypic hippocampal slice culture prepared from 8-week-old rat with vimentin as a NSC marker [37, 38]. VCP expression level was highly enriched in vimentin-immunoreactive stem cells in the SGZ in the presence of insulin, but its level significantly decreased in insulin-deprived slices (Fig. 1d). These results with organotypic slice cultures were well accordant with the data obtained from HCN cell culture, demonstrating the decrease of VCP protein level following insulin withdrawal in ex vivo and in vitro conditions. Despite the decrease of VCP protein levels in HCN cells and hippocampal slices, the levels of VCP mRNA expression were not changed by insulin withdrawal in HCN cells (Fig. 1e). These data suggest that VCP may be subject to post-translational regulation. To test whether VCP itself is a substrate for autophagic degradation or proteasome in insulin-deprived HCN cells, autophagic flux inhibitor, Bafilomycin A1 (BafA1) was treated following insulin withdrawal [39]. Interestingly, BafA1 (30 nM for 3 h) significantly prevented VCP degradation suggesting that autophagy may be responsible for degradation of VCP in insulin-deprived HCN cells (Fig. 1f).

### Pharmacological and genetic inactivation of VCP switched ACD to apoptosis in insulin-deprived HCN cells

**Fig. 2:**
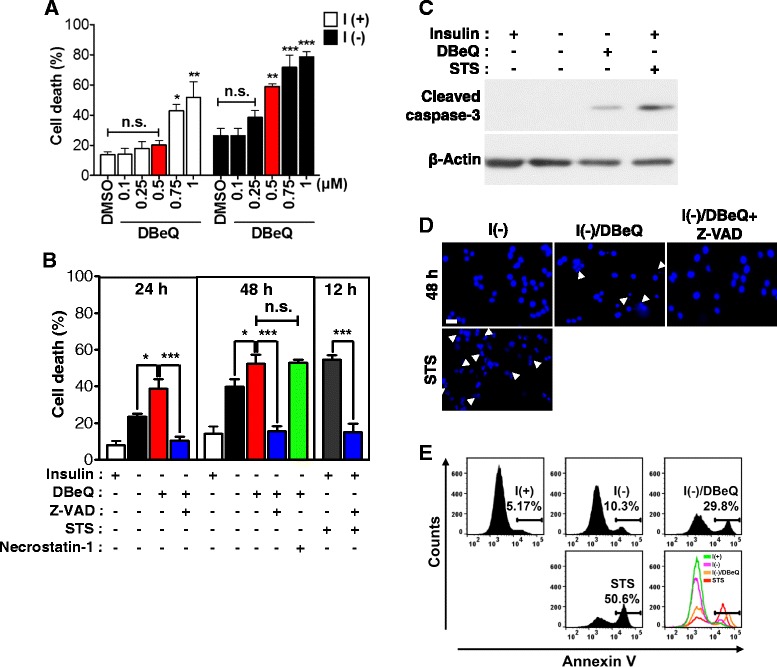
Pharmacological inhibition of VCP switched ACD to apoptosis in insulin-deprived HCN cells. **a** A VCP inhibitor, DBeQ (0.5 μM) markedly increased cell death following insulin withdrawal for 24 h, but without significant effect on I(+) HCN cells. Quantitative data were determined using One-way ANOVA followed by Tukey’s multiple comparison test. *p < 0.05, **p < 0.01, ***p < 0.001. n.s., not significant. **b** Z-VAD-FMK significantly prevented cell death induced by DBeQ in I(-). Staurosporine (STS, 1 μM for 12 h) was treated in I(+) HCN cells as a positive control for apoptosis induction. However, addition of necrostatin-1 (10 μM) up to 48 h did not decrease cell death in DBeQ-treated I(-) HCN cells. Quantitative data are represented as the mean ± SD by One-way ANOVA using Tukey’s multiple comparison test (*n* = 3). **p < 0.01, ***p < 0.001. n.s., not significant. **c** Caspase 3 was activated in DBeQ-treated I(-) HCN cells. **d** Nuclear condensation was observed by Hoechst staining, as indicated by *arrow*. Nuclear condensation was detected in DBeQ-treated I(-) HCN cells, but prevented by Z-VAD-FMK. Scale bar is 20 μm. **e** Apoptosis induction was confirmed by Annexin-V staining and FACS analysis for detection of the exposure of phosphatidylserine

VCP is known as a positive regulator in autophagosome maturation. To test whether VCP ATPase activity is required for ACD in HCN cells, HCN cells were treated with VCP ATPase activity inhibitor, *N*^*2*^*, N*^*4*^-dibenzylquinazoline-2,4-diamine (DBeQ) [40], in insulin-depleted media and cell death was measured. We tested a wide range of DBeQ concentrations both in I (+) and I (-) HCN cells and chose 0.5 μM concentration of DBeQ, based on marginal effect on cell death in I (+) but significant increase of cell death in I (-) HCN cells (Fig. 2a). Hereafter, this concentration of DBeQ has been used throughout this study. Since cell death in I(-) HCN cells was boosted by DBeQ, we wondered if this increase was due to the continuance of the default ACD or switch to different mode of PCD. To determine whether ACD was affected by DBeQ in I(-) HCN cells, the different kinds of PCD inhibitors were treated (Fig. 2b). Consistent with the absence of apoptotic indices, Z-VAD-FMK failed to reduce cell death in I(-) HCN cells (data not shown), which was well documented in our prior studies [13–15]. However, interestingly, Z-VAD-FMK significantly decreased cell death in DBeQ-treated I(-) HCN cells (Fig. 2b), suggesting that apoptosis was induced by VCP inhibition in I (-) HCN cells, which otherwise underwent genuine ACD. Staurosporine (STS), a common apoptosis inducer, was used as a positive control of apoptosis in I(+) HCN cells. STS-induced cell death was efficiently prevented by a broad-spectrum caspase inhibitor, Z-VAD-FMK (Fig. 2b). Also, necrostatin-1, a widely used necroptosis inhibitor [41], did not change cell death rate in DBeQ-treated I(-) HCN cells. This pharmacological inhibitor study suggests that VCP inhibition triggered a switch of cell death mode from ACD to apoptosis in I(-) HCN cells. Further analyses were performed to demonstrate the induction of apoptosis following VCP inhibition in I (-) HCN cells. Cleaved, active form of caspase 3 and chromatin condensation was observed in DBeQ-treated I(-) HCN cells (Fig. 2c, d). A marked increase in Annexin V positive staining indicating the derangement of phosphatidylserine distribution during apoptosis was also observed in DBeQ-treated I(-) HCN cells (Fig. 2e). Taken together, these data show that inhibition of VCP switches ACD to apoptosis in insulin-deprived HCN cells.

**Fig. 3:**
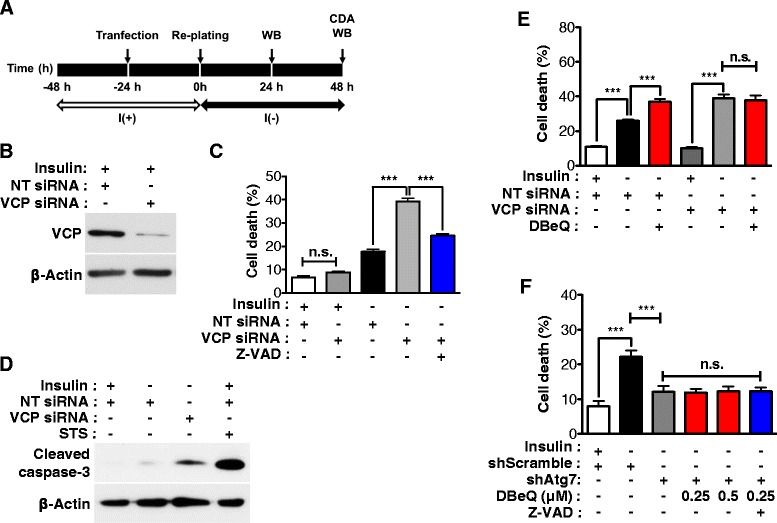
Genetic inhibition of VCP changed ACD to apoptosis in insulin-deprived HCN cells. **a** An experimental scheme for the knockdown of VCP. CDA, cell death assay; WB, Western blotting. **b** Knockdown of VCP was confirmed by Western blotting after 24 h treatment. NT, non-targeting. **c** VCP knockdown increased cell death and Z-VAD-FMK efficiently blocked an increase of cell death in I(-) HCN cells. Quantitative data are determined as the mean ± SD by One-way ANOVA followed by Tukey’s multiple comparison test (*n* = 3). ***p < 0.001. n.s., not significant. **d** Cleaved caspase 3 was detected in I(-) HCN cells following VCP knockdown at 48 h. **e** Cell death rate was not affected by DBeQ in I(-) HCN cells depleted of VCP. Quantitative data are determined as the mean ± SD by One-way ANOVA followed by Tukey’s multiple comparison test (*n* = 3). ***p < 0.001. n.s., not significant. **f** Atg7 knockdown decreased cell death in I(-) HCN cells. However, cell death rate was not altered by DBeQ or Z-VAD-FMK in I(-) HCN cells with stable knockdown of Atg7. Quantitative data are determined as the mean ± SD by One-way ANOVA followed by Tukey’s multiple comparison test (*n* = 3). ***p < 0.001. n.s., not significant

To further test whether genetic suppression of VCP expression also switches ACD to apoptosis, VCP-targeting small interfering RNA (siRNA) was transfected in HCN cells following the presented experimental scheme (Fig. 3a–b). In support of the results obtained with DBeQ treatment, knockdown of VCP did not alter cell death rate in I(+) condition, but significantly enhanced cell death in I(-) HCN cells (Fig. 3c). Augmented cell death in I(-) HCN cells by VCP knockdown was substantially diminished by Z-VAD-FMK (Fig. 3c) and accompanied by caspase-3 activation (Fig. 3d). Collectively, these data indicate that genetic knockdown of VCP expression induced a switch of PCD mode from ACD to apoptosis in I(-) HCN cells in a similar fashion as pharmacologic inhibition of VCP. However, it should be noted that VCP depletion or inactivation did not induce apoptosis at basal state in I(+) HCN cells, which is in sharp contrast with the previous studies reported in yeast, *Caenorhabditis. elegans*, HeLa, and several other mammalian cells [31, 42–49].

To exclude the possibility that DBeQ induces apoptosis independent of inhibiting VCP, we treated VCP-suppressed I(-) HCN cells with DBeQ. Unlike the elevation of cell death in NT siRNA-treated HCN cells, DBeQ failed to increase cell death in I(-) HCN cells depleted of VCP (Fig. 3e). These data suggest that DBeQ-induced apoptosis in I(-) condition was VCP-dependent. Also, to test whether inhibition of VCP by DBeQ was due to perturbation of non-autophagy related functions of VCP independently of autophagy, we stably knocked down Atg7 in HCN cells before DBeQ treatment. HCN cells were transduced with the lentivirus expressing Atg7-targeting shRNA and subject to puromycin selection. Atg7 knockdown significantly decreased ACD in I(-) HCN cells, consistent with our previous studies [14, 15]. Of particular interest, DBeQ failed to increase cell death in I(-) HCN cells depleted of Atg7, nor did Z-VAD-FMK affect cell death (Fig. 3f). These data strongly demonstrate that ACD is a prerequisite to DBeQ-induced switch to apoptosis in HCN cells and DBeQ-induced apoptosis proceeds following yet-to-be-discovered mechanism for interaction between ACD and apoptosis, but is not non-specific cytotoxic event.

### VCP inactivation reduced autophagy flux in insulin-deprived HCN cells

**Fig. 4:**
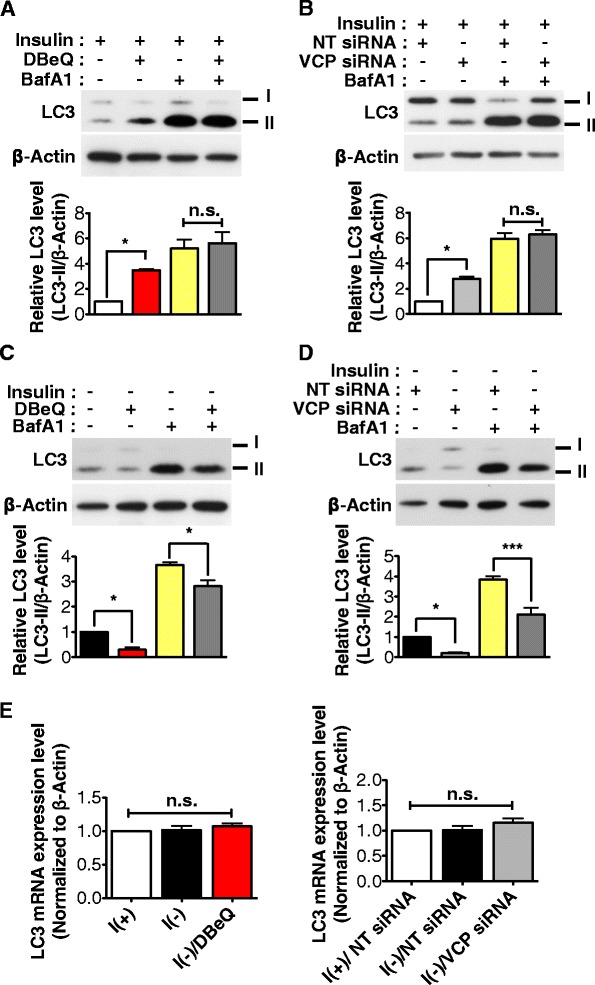
VCP inhibition reduced autophagic flux in insulin-deprived HCN cells. **a**-**b** DBeQ treatment (**a**) or VCP knockdown (**b**) induced a moderate increase in LC3-II in I(+) HCN cells. Blocking of autophagy using BafA1 gave rise to a significant increase of LC3-II with the same levels between BafA1 alone and BafA1/DBeQ or BafA1/VCP knockdown cells in I(+). Quantitative LC3-II levels were determined as the mean ± SD by One-way ANOVA followed by Tukey’s multiple comparison test (*n* = 3). **p < 0.01, ***p < 0.001. n.s., not significant. **c**-**d** DBeQ treatment (**c**) or VCP knockdown (**d**) led to a decrease in LC3-II in I(-) HCN cells. Blocking of autophagy using BafA1 gave rise to a significant increase of LC3-II, but the amount of accumulated LC3-II in BafA1/DBeQ or BafA1/VCP knockdown cells was significantly less than BafA1 alone in I(-). Quantitative LC3-II levels were determined as the mean ± SD by One-way ANOVA followed by Tukey’s multiple comparison test (*n* = 3). **p < 0.01, ***p < 0.001. n.s., not significant. **e** mRNA expression level of LC3 did not show different changes by VCP inactivation in I (-). Quantitative LC3 mRNA expression levels are represented as the mean ± SD (*n* = 3). Quantitative data was determined by Student’s t-test. *p < 0.05. n.s., not significant

Our findings that loss of VCP activity switched ACD to apoptosis suggest that VCP is required for the progress of ACD in I(-) HCN cells. However, it is not known whether VCP functions at the autophagosome maturation step, as in other studies [27–29], or plays new roles in the autophagy of HCN cells upon insulin withdrawal. To elucidate what role VCP may play in the progression of autophagy in HCN cells, we depleted VCP function by siRNA or using the same chemical inhibition approach of VCP activity and examined the autophagy flux rate. VCP inactivation by DBeQ or siRNA knockdown in I(+) HCN cells led to a modest increase in LC3-II level (Fig. 4a–b). On the other hand, DBeQ treatment or VCP siRNA knockdown in I(-) HCN cells substantially decreased LC3-II levels (Fig. 4c–d). When autophagy flux was blocked by addition of BafA1, there was a marked increase in the amount of LC3-II and they reached the same level between BafA1 control and BafA1/DBeQ or BafA1/VCP siRNA in I(+) HCN cells (Fig. 4a–b). In the case of I(-) HCN cells, BafA1 also increased LC3-II levels. However, interestingly, BafA1-induced accumulation of LC3-II was significantly less in BafA1/DBeQ or BafA1/VCP siRNA than BafA1 control in I(-) HCN cells (Fig. 4c–d).

An increase in LC3-II level may indicate increased formation of autophagosomes with high autophagy flux. Yet, impairment of autophagy flux due to blocking of autophagosome-lysosome fusion and failure of autophagosome maturation can also lead to an increase in LC3-II. Therefore, mild increase following DBeQ-treatment or VCP depletion in I(+) HCN cells can be due to either increase or impairment in autophagy flux. If an increase of LC3-II observed in VCP-depleted HCN cells in I(+) was due to high rate of autophagy flux, BafA1 treatment would lead to higher amount of LC3-II than BafA1 I(+) control cells. However, the amount of LC3-II was the same, suggesting that the overall autophagy flux rate remained the same regardless of VCP inactivation during the basal autophagy in I(+). Therefore, this modest increase in LC3-II by VCP depletion can be interpreted due to partial inhibition of autophagosome maturation with the yield of the same amount of LC3-II accumulation following full inhibition of autophagy flux by BafA1. On the other hand, complete blocking of autophagy flux by BafA1 disclosed considerably smaller amount of LC3-II in VCP depleted I(-) HCN cells than I(-) control, indicating reduced rate of autophagy flux. Therefore, a decrease in LC3-II by DBeQ or VCP siRNA before BafA1 treatment imply the similar decrease of autophagy flux rate in I(-) HCN cells. Quantitative analysis of LC3 mRNA level did not show significant change by VCP inactivation (Fig. 4e). Taken together, these data demonstrate that VCP positively regulates autophagosome maturation at basal autophagy in I(+), as reported by others. In contrast, VCP regulates overall autophagy flux rate in insulin-deprived HCN cells when autophagy activity is high.

**Fig. 5:**
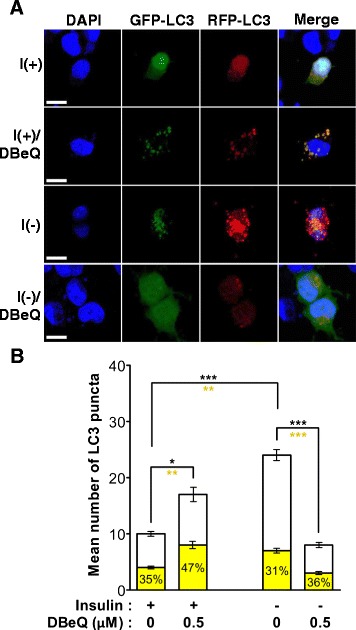
Differential regulation of autophagy flux by VCP in I(+) and I(-) HCN cells. **a** mRFP-GFP-LC3 was used for autopahgic flux assay. DBeQ treatment in I(+) led to an increase in the percentage of yellow puncta with no marked increase in the number of the total LC3 puncta, suggesting impairment of autophagosome maturation. DBeQ treatment in I(-) greatly reduced the number of the total LC3 puncta, but the percentage of yellow puncta remained similar between I(-) and I(-)/DBeQ conditions. Scale bar, 10 μm. **b** Quantitative *red and yellow* puncta numbers were determined as the mean ± SD by One-way ANOVA followed by Tukey’s multiple comparison test (*n* = 16–35). *Black and yellow asterisks* indicate the comparison of the total and yellow puncta, respectively. *p < 0.05, **p < 0.01, ***p < 0.001

Modest changes in the level of LC3-II may not be detected due to saturation of Western blotting signals. To further validate different roles of VCP for autophagy between I(+) and I(-) conditions, next, we adopted monomeric RFP-GFP tandem fluorescent LC3 (mRFP-GFP-LC3) and measured autophagy flux. Co-existence of GFP and RFP signals in autophagosomes before maturation and RFP signals in autolysosomes after maturation are based on the acid-sensitive quenching of EGFP protein. DBeQ treatment in I(+) increased the percentage of yellow puncta, but without significant increase in the total puncta number, suggesting attenuation of autophagosome maturation by DBeQ in I(+) HCN cells. On the other hand, LC3 puncta formation was strikingly increased in I(-) HCN cells and this increase was greatly reduced by DBeQ. However, the percentages of yellow puncta remained similar between I(-) and I(-)/DBeQ conditions, indicating that DBeQ inhibited autophagy induction rather than maturation step in I(-) HCN cells (Fig. 5).

### VCP is required for autophagy initiation signaling in insulin-deprived HCN cells

**Fig. 6:**
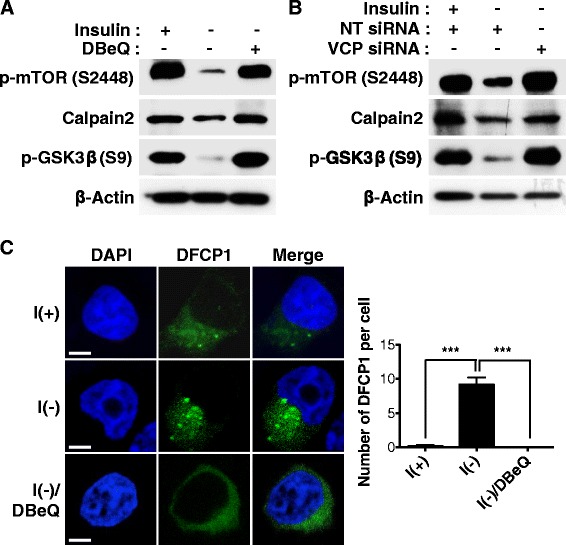
VCP is required for autophagy initiation signaling in HCN cells following insulin withdrawal. **a**-**b** Negative regulators of autophagy, mTOR and calpain 2 were suppressed in I(-), but reactivated by DBeQ treatment (**a**) or VCP knockdown (**b**). On the contrary, positive inducers of autophagy such as GSK-3β were activated following insulin withdrawal, but deactivated by DBeQ treatment (**a**) or VCP knockdown (**b**). **c** The high number of DFCP1 puncta observed in I(-) were greatly reduced by DBeQ treatment. The number of DFCP1 puncta was quantified with cut-off diameter of 0.5 μm. Scale bar, 5 μm. Quantitative DFCP1 puncta were determined as the mean ± SD by One-way ANOVA followed by Tukey’s multiple comparison test (*n* = 10). ***p < 0.001

Based on our results indicating the reduced autophagy level by VCP inactivation in I(-) HCN cells, we postulated that VCP may regulate autophagy initiation signaling in HCN cells following insulin withdrawal. Our previous study by Ha et al. demonstrated that glycogen synthase kinase 3β (GSK-3β) induced ACD in HCN cells following insulin withdrawal [15]. Both pharmacological and genetic inactivation of GSK-3β significantly decreased ACD, while activation of GSK-3β increased autophagic flux and caused more cell death without inducing apoptosis following insulin withdrawal. This report uncovered GSK-3β as one of the critical upstream signaling kinases for ACD in I(-) HCN cells. GSK-3β activation, as revealed by a decrease in the inhibitory phosphorylation of serine nine following insulin withdrawal, was significantly prevented by both pharmacological and genetic inactivation of VCP (Fig. 6a–b). Mammalian target of rapamycin (mTOR) is known for a nutrient sensor and negative regulator of autophagy [50]. mTOR activation in relation to autophagy suppression is reflected by various phosphorylation sites including serine 2448 in response to insulin or nutrient stimulation [51, 52]. S2448 of mTOR was dephosphorylated in I(-) HCN cells. However, its phosphorylation level was rescued by VCP inaction surprisingly even in the absence of insulin (Fig. 6a–b). In our previous report, we demonstrated that calpain 2 was degraded by UPS in I(-) HCN cells, but conditions leading to calpain accumulation and high calpain activity utterly switched the default ACD to apoptosis in I(-) HCN cells [14]. In accordance with the induction of apoptosis in VCP-inactivated I(-) HCN cells, calpain 2 level was also restored to much higher level than I(-) (Fig. 6a). Knockdown of VCP in I(+) induced mild changes in the phosphorylation level of mTOR (data not shown). Compared with dramatic recovery of mTOR phosphorylation by VCP knockdown in I(-), these data indicate that deficit in autophagy induction in I(-) HCN cells depleted of VCP is like a direct result of VCP inactivation specifically in I(-) condition.

Double FYVE-domain-containing protein 1 (DFCP1) interacts with phosphatidylinositol 3-phosphate (PtdIns(3)p), which is required for the early step of autophagosome formation. Therefore, DFCP1 is regarded as an autophagy initiation marker [53]. A transient transfection of DFCP1-GFP construct gave the high number of DFCP1 puncta in I(-) HCN cells, compared with the negligible number in I(+). However, DBeQ treatment strongly suppressed the formation of DFCP1 puncta, again suggesting that VCP activity is required for autophagy initiation in I (-) HCN cells (Fig. 6c). This novel role of VCP in autophagy initiation has not been reported before. Taken together, these data suggest that VCP contributes to the maturation of autophagosomes to autolysosomes at basal autophagy process under I(+) condition. However, when autophagy flux is intensified upon insulin withdrawal, VCP is critically involved in the initiation stage of autophagy, as well exemplified in insulin-deprived HCN cells, which undergoes high autophagy level and cell death.

## Discussion

In this report, we demonstrate that VCP is a key regulator of autophagy initiation signaling and a mediator of crosstalk between ACD and apoptosis in insulin-deprived HCN cells. The analysis of the phenotypes of VCP inhibition or knockdown revealed two important observations regarding autophagy process and cell death. First, VCP inactivation did not alter autophagy induction under normal condition when insulin was provided. Rather VCP was required for autophagosome maturation. On the other hand, VCP inhibition or depletion down-regulated autophagy induction signals in I(-) HCN cells. Second, our results indicate that ACD is the dominant mode of PCD in insulin-deprived HCN cells, but apoptosis can be an alternative route to cell death when autophagy level is subdued by VCP inhibition or depletion. However, unlike other studies, VCP inactivation itself, does not induce apoptosis in HCN cells under normal culture condition. Our data suggest that regulation of switch between ACD and apoptosis by VCP constitutes a unique regulatory module of cell death program when HCN cells are doomed to die following insulin withdrawal.

Our previous study revealed that calpain 2 inhibition and GSK-3β activation promoted ACD following insulin withdrawal HCN cells [14, 15]. In accordance with inhibition of ACD upon VCP inactivation in I(-) condition, there was significant increase in calpain 2 and inactive form of GSK-3β. GSK-3β and calpain 2 are anticipated as potential functional or physical interacting candidates of VCP in HCN cells.

**Fig. 7:**
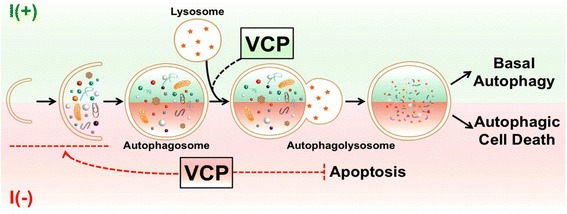
A schematic diagram illustrating the novel function of VCP in ACD and apoptosis in HCN cells following insulin withdrawal. HCN cells undergo autophagic cell death following insulin withdrawal. In basal autophagy, VCP positively regulates autophagosome maturation. However, following insulin withdrawal, VCP regulates autophagy initiation and the crosstalk between ACD to apoptosis

We propose the following model for distinct roles of VCP under different cellular stress conditions (Fig. 7). In I(+) cells, VCP ensures the maturation of autophagosomes and blocking of VCP activity impairs the late stage of autophagy flux at basal autophagy. When cells are encountered with high autophagy level following insulin withdrawal, depletion of VCP activity affects the early stage of autophagy flux and diminishes the autophagy initiating signals. Of note, our novel discovery also indicates another function of VCP in association with crosstalk between autophagy and apoptosis, as the inaction of VCP dramatically decreased autophagy initiation while the mode of cell death switched to apoptosis in insulin-deprived HCN cells. Further studies should clarify how VCP moves from a late stage to an early stage of autophagy under condition of insulin withdrawal. Another important direction of future research will be to understand how VCP affects the decision of cell death mode between ACD and apoptosis, and the molecular details regulating this VCP activity during ACD.

Autophagy and apoptosis plays an essential role in development and survival of NSCs. Basal autophagy is essential to protect cells against stress conditions by degrading damaged organelles and proteins. However, excessive autophagy can cause ACD. The abnormal regulation of autophagy, such as too little autophagy or too much autophagy and ACD, plays an important role in aging and neurodegenerative diseases [54–58]. The efforts of understanding the molecular mechanisms of ACD have continued in recent years. Knowledge on the molecular mechanisms of ACD and its interconnection with apoptosis will contribute to the strategic design aimed at protecting NSCs from degenerative conditions and cell death. High expression level of VCP in HCN cells in vivo and in vitro and its interesting role in regulation of autophagy and PCD of HCN cells warranties further detailed study on the role of VCP in the function of NSCs to understand the sophisticated molecular mechanisms of PCD in NSCs.

## Methods

### Antibodies and reagents

The following antibodies and reagents were used: VCP antibody was purchased from Cell signaling Technology (Danvers, MA, USA) or Abcam (Cambridge, United Kingdom). β-actin, calpain2, GSK-3β (S9), mTOR (S2448) and cleaved caspase-3 antibodies were purchased from Cell Signaling Technology; LC3B antibody from Sigma-Aldrich (St. Louis, MO, USA); MAP2 antibody from Abcam; GFAP and Vimentin antibodies from Millipore (Billerica, MA, USA). DBeQ, staurosporine, lactacystin and BafA1 were purchased from Sigma-Aldrich, Z-VAD-FMK from R&D Systems (Minneapolis, MN, USA), necrostatin-1 from Enzo Life Sciences (Farmingdale, NY, USA). They were diluted in dimethyl sulfoxide at appropriate concentrations.

### Cell culture

HCN cells were grown as described previously by Chung et al (2015) [14].

### Plasmids, siRNA and transfection

HCN cells were seeded a cell density of 1.5 × 10^5^ cells/mL. HCN cells were suspended in 100 μl of Nucleofector Kit solution and were transfected with siRNA specific for rat VCP (Dharmacon, Lafayette, CO, USA) by using a Nucleofector Kit (Lonza, Basel, Switzerland) according to the manufacturer’s instructions with minor modification. After the nucleofection, the cells were incubated for 1 day and re-seeded in plates according to the experimental designs. pMXs-puro GFP-DFCP1 for mouse was purchased from Addgene (Cambridge, MA, USA). pMXs-puro GFP-DFCP1 and mRFP-GFP-LC3 constructs were transfected by Lipofectamine 2000 (Invitrogen, Carlsbad, CA, USA) according to the manufacturer’s instructions. Transfection was performed in culture medium without penicillin/streptomycin for 4 h. After, HCN cells were incubated with culture medium.

### Cell death assay

Cell death was measured in a 96-well plate at a cell density of 1 × 10^5^ cells/mL. HCN cells were stained with diluted Hoechst 33342 (Invitrogen, Carlsbad, CA, USA) and propidium iodide (PI; Sigma-Aldrich) (1 % volume of media in the well, final 1/2000 and 1/1000 dilution) for 20 min at 37 °C. Cell counting was performed under a fluorescence microscope and was analyzed using Pixcavator IA.

### Western blotting

HCN cells were harvested and lysed in radioimmunoprecipitation assay buffer (RIPA buffer, Sigma-Aldrich) with 1× protease cocktail inhibitors (Thermo Scientific, Waltham, MA, USA) and 1× phosphatase cocktail inhibitors (Thermo Scientific), 1 mM dithiothreitol and 1 mM phenylmethylsulfonyl fluoride for 30 min on ice. After centrifugation (16,100 × g, 10 min), protein concentrations were measured using the BCA protein assay reagent (Thermo Scientific). Proteins were loaded into the gel and electrotransfered to polyvinylidene difluoride (PVDF) membrane with a semi-dry electrophoretic transfer cell (Bio-Rad, Richmond, CA). Membranes were blocked for 1 h at room temperature in a blocking solution consisting of 5 % nonfat dry milk, 0.1 % Tween 20, and Tris-buffered saline (TBST). The membranes were then incubated overnight with the primary antibodies diluted in the blocking solution and washed three times for 10 min. The membranes were incubated for 1 h at room temperature with peroxidase-conjugated secondary antibodies diluted in blocking solution. After washing, protein expression was detected by using a chemiluminescence detection kit (Thermo Scientific).

### Immunohistochemistry

All procedures for the care and use of laboratory animals were approved by the Institutional Animal Care and Use Committee (IACUC) at Daegu Gyeongbuk Institute of Science and Technology (DGIST). The 8-week-old rats were perfused with 1× PBS and 4 % PFA. After dissection, the post-fixation was performed with 4 % PFA for 12 h. The brain was immersed in 30 % sucrose in PBS at 4 °C until it sank. The brain was embedded in OCT compound and frozen on dry ice. The brain was cut into 40 μm serial fee-floating coronal sections. The brain sections were washed with 1× PBS three times for 10 min. Blocking was performed for 1 h into 5 % normal donkey serum (NDS) (Jackson ImmunoResearch Laboratories Inc., West Grove, PA, USA) with 1 % BSA and 0.3 % Triton X-100. The brain sections were incubated with primary antibody diluted in 5 % NDS and 0.3 % Triton X-100 for 18 h at 4 °C. After washing with 1× PBS, the brain sections were incubated with secondary antibody diluted in 5 % NDS and 0.3 % Triton X-100 for 2 h at room temperature in dark. Hoechst 33342 (Invitrogen) (1/10000 dilution) was stained for 10 min at room temperature. After washing, the brain sections were mounted on slide glass. The imaging was processed under laser scanning microscopes 700 (Zeiss, Oberkochen, Germany).

### Quantitative reverse transcription polymerase chain reaction (qRT-PCR)

Total RNA was isolated using the ImProm-II Reverse Transcriptase kit (Promega, Madison, WI, USA) and cDNA was synthesized. qRT-PCR was performed with the CFX96 Real-Time System (Bio-Rad) and TOPreal™ qPCR 2× PreMIX (SYBR Green with low ROX) (Enzynomics, Daejeon, Republic of Korea). β-actin was used as the reference gene for normalization. Primers used for VCP, LC3 and β-actin were as follows: VCP forward, 5′- AGAGCAACCTTCGTAAAGCC-3′, and reverse, 5′-AACAACTGAGACACGATGCG-3′; LC3 forward 5′- AGTGGAAGATGTCCGGCTCA-3′, and reverse, 5′- GCTTCTCACCCTTGTATCGCT-3′; β-actin forward, 5′- GTCCACCCGCGAGTACAACCTT-3′, and reverse, 5′- TTGCACATGCCGGAGCCGTT-3′.

### Annexin V staining and flow cytometry analysis

Cells were trypsinized and harvested in phosphate-buffered saline (PBS). Centrifuged cells were washed in PBS and re-suspended with 100 μl of 1× Annexin V binding buffer (10 mM HEPES, pH 7.4), 140 mM NaCl, 2.5 mM CaCl_2_). For labeling, Annexin V-FITC (BD Biosciences, San Jose, CA, USA) was added and incubated for 15 min at room temperature. After the incubation, 400 μl of Annexin V binding buffer was added to dilute the sample. Samples were kept in ice, protected from light, and used for the analysis within an hour. Analysis was taken by flow cytometry using the Accuri C6 Flow Cytometer System (BD Biosciences) according to the manufacturer’s instructions.

### Embryonic primary hippocampal neuronal culture

Pregnant rats were purchased from Koatech (Namyangju-si, Gyeonggi-Do, Republic of Korea) and primary rat hippocampal neurons were prepared and cultured from rat embryos at day 17 (E-17). In brief, E-17 hippocampi were dissected and dissociated in a serum-free environment, and maintained in a serum-containing medium. Medium consists of Neurobasal medium (Gibco) supplemented with 100 U/ml penicillin (HyClone, Logan, UT, USA), 100 μg/ml streptomycin (HyClone), 2 mM L-glutamine (Sigma-Aldrich), and 2 % B27 supplement (Gibco). Three to four days after plating, medium was changed by one-third of the volume per well with fresh medium, which contained cytosine β-D-arabinofuranoside (Sigma-Aldrich) to a final concentration of 2 μM. The culture continued for up to 2 to 3 weeks until used for the experiment with change of medium by one-third of the volume every 3–4 days.

### Hippocampus slice culture

Organotypic hippocampal slices were prepared according to the method described by Simoni and Yu [59] slight modifications due to experimental designs. Adult female Sprague Dawley rats (7–8 weeks of age from Koatech) were decapitated following all institutional and ethical regulations regarding animal handling. Brains were rapidly removed and placed into a ice-cold slicing medium composed of 0.02 mM HEPES in Earle’s Balanced Salt Solution (EBSS; Welgene, Republic of Korea). Hippocampi were dissected out in fresh chilled slicing medium. Extracted hippocampi were then placed on the Teflon stage of a manual tissue slice chopper (Leica, Wetzlar, Germany) for coronal sectioning at 350 μm. Four hippocampal slices were seeded onto each Millicell culture plate insert (Millipore) in 6-well plates. After a 2-day stabilization period, hippocampal slices were deprived of insulin for 48 h prior to further experimental use.

### Statistical analysis

All data from at least three independent experiments were analyzed as mean ± standard deviation (SD). Statistical significance was determined by the unpaired Student’s t-test or one-way analysis of variance (ANOVA) by Tukey’s multiple comparison tests using Graphpad Prism (GraphPad Software, San Diego, CA, USA).

## Conclusions

We demonstrate that VCP plays a new role by inducing autophagy initiation in insulin-deprived HCN cells. Of particular interest, VCP inactivation in insulin-deprived HCN cells swithces the mode of PCD from ACD to apoptosis. Our study will contribute to understanding the novel roles of VCP in regulation of autophagy and its interaction with apoptosis in the NSCs.

## Abbreviations

BafA1: Bafilomycin A1
DBeQ: *N*^*2*^*, N*^*4*^-dibenzylquinazoline-2,4-diamine
GSK-3β: glycogen synthase kinase
HCN cell: hippocampal neural stem cell
LC3- II: the type II of microtubule-associated protein light chain 3
NSC: neural stem cell
NT siRNA: non-targeted siRNA
PBS: phosphate buffed saline
PCD: programmed cell death
PI: propidium iodide
siRNA: small interfering RNA
STS: starousporine
VCP: valosin-containing protein

## References

[1] Fuchs Y, Steller H (2011). Programmed cell death in animal development and disease. Cell.

[2] Clarke PG (1990). Developmental cell death: morphological diversity and multiple mechanisms. Anat Embryol.

[3] Kerr JF, Wyllie AH, Currie AR (1972). Apoptosis: a basic biological phenomenon with wide-ranging implications in tissue kinetics. Br J Cancer.

[4] Shintani T, Klionsky DJ (2004). Autophagy in health and disease: a double-edged sword. Science.

[5] Wang CW, Klionsky DJ (2003). The molecular mechanism of autophagy. Mol Med.

[6] Lockshin RA, Zakeri Z (2001). Programmed cell death and apoptosis: origins of the theory. Nat Rev Mol Cell Biol.

[7] Cuervo AM (2004). Autophagy: in sickness and in health. Trends Cell Biol.

[8] Mizushima N, Komatsu M (2011). Autophagy: renovation of cells and tissues. Cell.

[9] Berry DL, Baehrecke EH (2007). Growth arrest and autophagy are required for salivary gland cell degradation in Drosophila. Cell.

[10] Batlevi Y, Martin DN, Pandey UB, Simon CR, Powers CM, Taylor JP (2010). Dynein light chain 1 is required for autophagy, protein clearance, and cell death in Drosophila. Proc Natl Acad Sci U S A.

[11] Wen YD, Sheng R, Zhang LS, Han R, Zhang X, Zhang XD (2008). Neuronal injury in rat model of permanent focal cerebral ischemia is associated with activation of autophagic and lysosomal pathways. Autophagy.

[12] Zhu JH, Horbinski C, Guo F, Watkins S, Uchiyama Y, Chu CT (2007). Regulation of autophagy by extracellular signal-regulated protein kinases during 1-methyl-4-phenylpyridinium-induced cell death. Am J Pathol.

[13] Yu SW, Baek SH, Brennan RT, Bradley CJ, Park SK, Lee YS (2008). Autophagic death of adult hippocampal neural stem cells following insulin withdrawal. Stem Cells.

[14] Chung KM, Park H, Jung S, Ha S, Yoo SJ, Woo H (2015). Calpain Determines the Propensity of Adult Hippocampal Neural Stem Cells to Autophagic Cell Death Following Insulin Withdrawal. Stem Cells.

[15] Ha S, Ryu HY, Chung KM, Baek SH, Kim EK, Yu SW (2015). Regulation of autophagic cell death by glycogen synthase kinase-3beta in adult hippocampal neural stem cells following insulin withdrawal. Mol Brain.

[16] Shen HM, Codogno P (2011). Autophagic cell death: Loch Ness monster or endangered species?. Autophagy.

[17] Meyer H, Bug M, Bremer S (2012). Emerging functions of the VCP/p97 AAA-ATPase in the ubiquitin system. Nat Cell Biol.

[18] Pye VE, Dreveny I, Briggs LC, Sands C, Beuron F, Zhang X (2006). Going through the motions: the ATPase cycle of p97. J Struct Biol.

[19] DeLaBarre B, Brunger AT (2005). Nucleotide dependent motion and mechanism of action of p97/VCP. J Mol Biol.

[20] Schuberth C, Buchberger A (2008). UBX domain proteins: major regulators of the AAA ATPase Cdc48/p97. Cell Mol Life Sci.

[21] Yeung HO, Kloppsteck P, Niwa H, Isaacson RL, Matthews S, Zhang X (2008). Insights into adaptor binding to the AAA protein p97. Biochem Soc Trans.

[22] Kondo H, Rabouille C, Newman R, Levine TP, Pappin D, Freemont P (1997). p47 is a cofactor for p97-mediated membrane fusion. Nature.

[23] Meyer HH, Shorter JG, Seemann J, Pappin D, Warren G (2000). A complex of mammalian ufd1 and npl4 links the AAA-ATPase, p97, to ubiquitin and nuclear transport pathways. EMBO J.

[24] Wang Y, Satoh A, Warren G, Meyer HH (2004). VCIP135 acts as a deubiquitinating enzyme during p97-p47-mediated reassembly of mitotic Golgi fragments. J Cell Biol.

[25] Schuberth C, Buchberger A (2005). Membrane-bound Ubx2 recruits Cdc48 to ubiquitin ligases and their substrates to ensure efficient ER-associated protein degradation. Nat Cell Biol.

[26] Stolz A, Hilt W, Buchberger A, Wolf DH (2011). Cdc48: a power machine in protein degradation. Trends Biochem Sci.

[27] Ju JS, Fuentealba RA, Miller SE, Jackson E, Piwnica-Worms D, Baloh RH (2009). Valosin-containing protein (VCP) is required for autophagy and is disrupted in VCP disease. J Cell Biol.

[28] Tresse E, Salomons FA, Vesa J, Bott LC, Kimonis V, Yao TP (2010). VCP/p97 is essential for maturation of ubiquitin-containing autophagosomes and this function is impaired by mutations that cause IBMPFD. Autophagy.

[29] Seguin SJ, Morelli FF, Vinet J, Amore D, De Biasi S, Poletti A (2014). Inhibition of autophagy, lysosome and VCP function impairs stress granule assembly. Cell Death Differ.

[30] Watts GD, Wymer J, Kovach MJ, Mehta SG, Mumm S, Darvish D (2004). Inclusion body myopathy associated with Paget disease of bone and frontotemporal dementia is caused by mutant valosin-containing protein. Nat Genet.

[31] Braun RJ, Zischka H (2008). Mechanisms of Cdc48/VCP-mediated cell death: from yeast apoptosis to human disease. Biochim Biophys Acta.

[32] Liu Y, Namba T, Liu J, Suzuki R, Shioda S, Seki T (2010). Glial fibrillary acidic protein-expressing neural progenitors give rise to immature neurons via early intermediate progenitors expressing both glial fibrillary acidic protein and neuronal markers in the adult hippocampus. Neuroscience.

[33] Bloom GS, Vallee RB (1983). Association of microtubule-associated protein 2 (MAP 2) with microtubules and intermediate filaments in cultured brain cells. J Cell Biol.

[34] Kabeya Y, Mizushima N, Ueno T, Yamamoto A, Kirisako T, Noda T (2000). LC3, a mammalian homologue of yeast Apg8p, is localized in autophagosome membranes after processing. EMBO J.

[35] Mizushima N, Yoshimori T (2007). How to interpret LC3 immunoblotting. Autophagy.

[36] Jiang P, Mizushima N (2015). LC3- and p62-based biochemical methods for the analysis of autophagy progression in mammalian cells. Methods.

[37] Kim M, Habiba A, Doherty JM, Mills JC, Mercer RW, Huettner JE (2009). Regulation of mouse embryonic stem cell neural differentiation by retinoic acid. Dev Biol.

[38] Foret MR, Sandstrom RS, Rhodes CT, Wang Y, Berger MS, Lin CH (2014). Molecular targets of chromatin repressive mark H3K9me3 in primate progenitor cells within adult neurogenic niches. Front Genet.

[39] Klionsky DJ, Elazar Z, Seglen PO, Rubinsztein DC (2008). Does bafilomycin A1 block the fusion of autophagosomes with lysosomes?. Autophagy.

[40] Chou TF, Brown SJ, Minond D, Nordin BE, Li K, Jones AC (2011). Reversible inhibitor of p97, DBeQ, impairs both ubiquitin-dependent and autophagic protein clearance pathways. Proc Natl Acad Sci U S A.

[41] Degterev A, Hitomi J, Germscheid M, Ch’en IL, Korkina O, Teng X (2008). Identification of RIP1 kinase as a specific cellular target of necrostatins. Nat Chem Biol.

[42] Noguchi M, Takata T, Kimura Y, Manno A, Murakami K, Koike M (2005). ATPase activity of p97/valosin-containing protein is regulated by oxidative modification of the evolutionally conserved cysteine 522 residue in Walker A motif. J Biol Chem.

[43] Wojcik C, Yano M, DeMartino GN (2004). RNA interference of valosin-containing protein (VCP/p97) reveals multiple cellular roles linked to ubiquitin/proteasome-dependent proteolysis. J Cell Sci.

[44] Rao RV, Castro-Obregon S, Frankowski H, Schuler M, Stoka V, del Rio G (2002). Coupling endoplasmic reticulum stress to the cell death program. An Apaf-1-independent intrinsic pathway. J Biol Chem.

[45] Rao RV, Ellerby HM, Bredesen DE (2004). Coupling endoplasmic reticulum stress to the cell death program. Cell Death Differ.

[46] Vij N, Fang S, Zeitlin PL (2006). Selective inhibition of endoplasmic reticulum-associated degradation rescues DeltaF508-cystic fibrosis transmembrane regulator and suppresses interleukin-8 levels: therapeutic implications. J Biol Chem.

[47] Shirogane T, Fukada T, Muller JM, Shima DT, Hibi M, Hirano T (1999). Synergistic roles for Pim-1 and c-Myc in STAT3-mediated cell cycle progression and antiapoptosis. Immunity.

[48] Madeo F, Schlauer J, Frohlich KU (1997). Identification of the regions of porcine VCP preventing its function in Saccharomyces cerevisiae. Gene.

[49] Wu D, Chen PJ, Chen S, Hu Y, Nunez G, Ellis RE (1999). C. elegans MAC-1, an essential member of the AAA family of ATPases, can bind CED-4 and prevent cell death. Development.

[50] Jung CH, Ro SH, Cao J, Otto NM, Kim DH (2010). mTOR regulation of autophagy. FEBS Lett.

[51] Rosner M, Siegel N, Valli A, Fuchs C, Hengstschlager M (2010). mTOR phosphorylated at S2448 binds to raptor and rictor. Amino Acids.

[52] Watanabe R, Wei L, Huang J (2011). mTOR signaling, function, novel inhibitors, and therapeutic targets. J Nucl Med.

[53] Axe EL, Walker SA, Manifava M, Chandra P, Roderick HL, Habermann A (2008). Autophagosome formation from membrane compartments enriched in phosphatidylinositol 3-phosphate and dynamically connected to the endoplasmic reticulum. J Cell Biol.

[54] Kegel KB, Kim M, Sapp E, McIntyre C, Castano JG, Aronin N (2000). Huntingtin expression stimulates endosomal-lysosomal activity, endosome tubulation, and autophagy. J Neurosci.

[55] Yue Z, Horton A, Bravin M, DeJager PL, Selimi F, Heintz N (2002). A novel protein complex linking the delta 2 glutamate receptor and autophagy: implications for neurodegeneration in lurcher mice. Neuron.

[56] Stefanis L, Larsen KE, Rideout HJ, Sulzer D, Greene LA (2001). Expression of A53T mutant but not wild-type alpha-synuclein in PC12 cells induces alterations of the ubiquitin-dependent degradation system, loss of dopamine release, and autophagic cell death. J Neurosci.

[57] Wolfe DM, Lee JH, Kumar A, Lee S, Orenstein SJ, Nixon RA (2013). Autophagy failure in Alzheimer’s disease and the role of defective lysosomal acidification. Eur J Neurosci.

[58] Nixon RA, Yang DS (2011). Autophagy failure in Alzheimer’s disease--locating the primary defect. Neurobiol Dis.

[59] De Simoni A, Yu LM (2006). Preparation of organotypic hippocampal slice cultures: interface method. Nat Protoc.

